# Emission of airborne nanoparticles from electric motors of household appliances

**DOI:** 10.1007/s11869-025-01744-1

**Published:** 2025-05-13

**Authors:** Yevgen Nazarenko, Elliot Zolfaghar, Devendra Pal, Léa Quellard, Parisa A. Ariya

**Affiliations:** 1 Department of Environmental and Public Health Sciences, University of Cincinnati, 160 Panzeca Way, Cincinnati, OH 45267 USA; 2 Department of Chemistry, McGill University, 801 Sherbrooke Street West, Montréal, QC H3A 2K6 Canada; 3 Department of Atmospheric and Oceanic Sciences, McGill University, 805 Sherbrooke Street West, Montreal, QC H3A 0B9 Canada

**Keywords:** Nanoaerosols, Nanoparticles, Ultrafine particles, Electric motors, Indoor air, Air pollution

## Abstract

Nanoparticulate (ultrafine particle) indoor air pollution is an emerging concern. Evidence points to airborne nanoparticles’ potential adverse effects, including the impact on blood pressure, the pulmonary and cardiovascular systems, cognitive performance, oxidative stress, allergen sensitization, and inflammation. Nanoparticles originate from various sources. However, no study to date investigated emissions of nanoparticles and fine particles from electric motors in household appliances, ubiquitous indoors. This study fills this knowledge gap with an investigation of incidental emission of aerosol particles from seven electric motors taken from household appliances. The appliances were made by several different manufacturers and tested at the respective appliance’s maximum and minimum power settings as intended for use by the consumer. Aerosols were continuously measured and characterized during 24-hour measurement periods using a NanoScan scanning mobility particle sizer (SMPS). Two of the seven motors (AP B and AP G) emitted very few particles, with an average total number concentration below 10 cm^− 3^. One electric motor (AP E) emitted over 170 times more aerosol particles at the maximum power setting compared to the minimum power setting. Another motor (AP A) had the highest emission of all motors at both the minimum and the maximum power settings. The total number concentration of aerosol particles exceeded 2700 cm^− 3^ and 3900 cm^− 3^ when operating two of the investigated electric motors (AP A and AP E, respectively) at the maximum power setting. We recommend that manufacturers of electric motors and household appliances test fine and ultrafine aerosol particle emissions from their products and address the problem. The design of household appliances equipped with electric motors should consider low-aerosol-emission motors and/or the installation of high-efficiency air filters in the motor air cooling duct(s) downstream of the electric motors.

**Supplementary Information:**

The online version contains supplementary material available at 10.1007/s11869-025-01744-1.

## Introduction

Indoor air pollution is emerging as a significant threat to human health, considering that most individuals spend nearly all their lives in indoor environments (Klepeis et al. 2001; Ageel et al. 2022; U.S.EPA 2024). Indoor air pollution originates both indoors and outdoors. Outdoor air pollutants that originate from sources such as traffic, industry, construction, and wildfires infiltrate indoors and increase exposure to airborne pollutants in residential, commercial, industrial and other buildings (Thompson et al. 1973; Chen and Zhao 2011), adding to the sources of air pollutants indoors (World Health Organization (WHO) 2010). Concentrations of many air pollutants indoors often exceed those outdoors due to indoor sources (Blondeau et al. 2005; Zhu et al. 2005). Most homes still rely only on natural ventilation through open windows and infiltration through the building envelope (Morawska et al. 2020; Ageel et al. 2022; Fawaier and Bokor 2022). This presents a problem in a climate where natural ventilation is minimized to prevent loss of heat or energy during the air cooling season, especially in buildings that have been winterized (Emmerich et al. 2017), so pollutants from sources indoors are trapped and are not adequately ventilated out of many indoor spaces. The problem of indoor air pollution due to inadequate ventilation is also an environmental justice issue because many vulnerable people live, study and work in poorly ventilated homes with significant sources of indoor and infiltrated outdoor air pollution: as an example, this problem has been identified in Canadian Indigenous people’s communities (Ghoshdastidar et al. 2018). Indoor concentrations of nanoparticles (Wallace and Ott 2010; Ghoshdastidar et al. 2018) and certain bioaerosols (Kalogerakis et al. 2005; Lee et al. 2006) in Indigenous people’s residences have been measured to substantially exceed their concentrations outdoors.

Aerosol particles in indoor air range in size from smaller than 10 nm to as large as several dozen micrometers. Particulate matter (PM) is categorized, studied, and regulated by size fractions. The smallest particles in the air are nanoparticles, also called nanoaerosols or ultrafine particles (UFPs). Nanoparticles are most often defined as particles with a diameter smaller than roughly 100 nm (PM_0.1_). In indoor and outdoor air, nanoparticles are typically numerous compared to the abundance of fine, coarse, and supercoarse particles. PM is more toxic per unit mass as the size of aerosol particles decreases (Kelly and Fussell 2012). Thus, nanoparticles should be measured as aerosol surface area or, preferably, using a number-based metric (Manigrasso et al. 2017). The small size of nanoparticles enables them to be readily inhaled into the human respiratory system, and a higher proportion of them reach and deposit in the tracheobronchial and the alveolar regions compared to fine, coarse, and supercoarse particles (Lu et al. 2014). In the respiratory system, nanoparticles’ expansive surface area can enhance the rate of bloodstream penetration in the lungs, potentially heightening the toxicity of nanoparticles as they migrate through the body with the blood (Lu et al. 2014; Abdillah and Wang 2023). It is harder to measure the concentration of nanoparticles in the air compared to measuring larger particles, notably because the instruments for measuring them are substantially more expensive and require greater technical skills to operate and maintain, so measurements of nanoparticles indoors are scarce, and relevant health studies have been limited.

Electric motors often generate significant numbers of nanoparticles, a phenomenon that merits attention due to the potential impact on indoor air quality. In one study, particle emission rates as high as 7.47 × 10^7^ particles/s were measured (Wang et al. 2015). The particles, mostly below 100 nm in diameter, were generated from spark discharges inside the motors. The study identified copper and organic compounds as major components of the emitted particles (Wang et al. 2015). Findings demonstrate that electric universal motors, particularly when subjected to phase angle modulation for power control, tend to emit nanoparticles primarily composed of copper or graphite (Szymczak et al. 2007). Spikes in the concentration of emitted particles, predominantly falling within the nanoparticle size range, can increase nanoparticle concentrations by 5 − 12% above background during motor operation (Manigrasso et al. 2017).

Here, we investigated the hypothesis that electric motors in household appliances emit nanoparticles into indoor air. To our knowledge, this is the first study investigating household appliance electric motors, discovering an important source of nanoparticles indoors.

We report our findings of nanoparticle emission by seven different electric motors from household appliances produced by different manufacturers, including aerosol size distributions and number densities of particles emitted during the operation of the motors. Aerosol particle size distribution determines aerosol dynamics and deposition in different regions of the human respiratory system (Chen et al. 2016; Finlay and Darquenne 2020). Therefore, in this study, we present both aerosol particle size distribution and the total number concentration of aerosol particles in each segment of the aerosol particle size spectrum, which is critically important for human inhalation exposure characterization.

Emission by electric motors in household appliances is a significant non-combustion source of nanoparticles in indoor air. With this study, we draw the attention of the designers and manufacturers of household appliances and electric motors for household appliances to consider the level of aerosol nanoparticle emissions in the design of electric motors in household appliances.

## Materials and methods

The electric motors of each tested appliance (AP) were labeled with code names. Specific information about the tested appliances and motors is not disclosed due to confidentiality agreements and accessibility limitations, but they came from a variety of manufacturers. Each motor was tested under conditions prescribed by the manufacturer, ensuring relevance to real-world usage scenarios. The aerosol particles emitted by the electric motors were analyzed quantitatively and reported as aerosol number concentration, i.e., the particle number counts per cubic centimeter (#/cm^3^ or cm^− 3^), within the 13 measurement size channels of the NanoScan scanning mobility particle sizer (SMPS) instrument (Table S1) (TSI Incorporated 2013). The number of particles within the size channels is given as a count distribution. The geometric standard deviation (GSD) characterizes the spread of the distribution, the median represents the central tendency, and the mode represents the most abundant aerosol particle size in each aerosol size distribution.

**Fig. 1:**
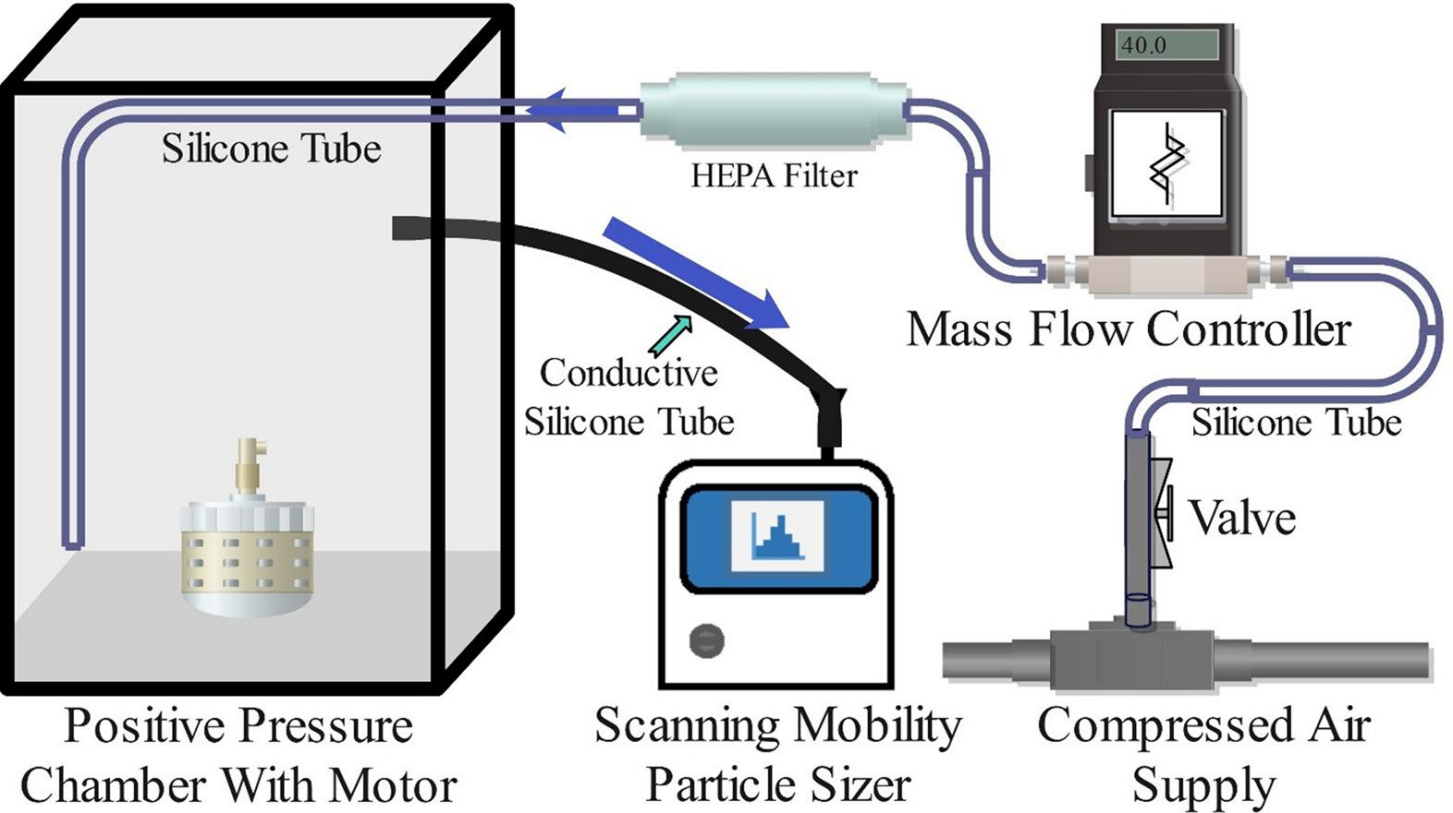
Schematic of the Positive-Pressure Chamber Setup for the Investigation of Aerosol Particle Emission from Electric Motors. The NanoScan SMPS real-time aerosol sizer and the metered clean air delivery system are shown. One of the investigated electric motors is depicted inside the chamber

A positive-pressure chamber setup was constructed for the investigation of aerosol particle emissions from 7 electric motors (Fig. 1).

The chamber had poly(methyl methacrylate) walls with inner dimensions of 560 mm × 560 mm × 760 mm. The front wall of the chamber was removable and only partially sealed to allow the air injected into the chamber to constantly escape. Clean air was constantly injected into the chamber at 40.0 SL/min, controlled by a mass flow controller, model C100L-DD-3-OV1-SV1-PV2-V1-S0-C10 (Sierra Instruments Inc., Monterey, CA, USA). This airstream was filtered through a large HEPA (High-Efficiency Particulate Arrestance) filter, Whatman HEPA-CAP 150 (Little Chalfont, Buckinghamshire, UK). Thus, the chamber was under slight positive pressure with a constant 40 SL/min through-flow. The constant flow of clean air facilitated the investigation of the total number of particles emitted by each motor per unit time into a known unit mass of air. This design ensured a nearly aerosol-particle-free environment inside the chamber when no motor was operated inside the chamber and no aerosol particles were emitted. The nearly particle-free environment was confirmed (Figure S1) by the real-time aerosol measurement instrument, NanoScan SMPS, Model 3910 (TSI, Inc., Shoreview, MN, USA), also used for measuring motor emissions as described in detail further down.

To test aerosol particle emissions from each electric motor, a given household appliance subject to investigation was stripped of all components that could be removed, apart from the electric motor with the attached power control circuit. Each such unit was cleaned and placed in the test chamber one unit at a time for each experiment. Before each experiment, a background measurement was taken for the duration of 30 to 60 min until the final total number concentration of aerosols, added up across all size channels, dropped to below 2 cm^− 3^.

The NanoScan SMPS continuously sampled the air in the chamber housing one electric motor at a time. The NanoScan SMPS is a particle electrical mobility-based instrument with a radial differential mobility analyzer (RDMA) coupled with an internal condensation particle counter (CPC). The measurement size range of the instrument is 10 nm to 420 nm, with the D_50_ cut-off point of the inlet cyclone at roughly 550 nm and a sampling time resolution of 60 s for size distribution measurements. The inlet sampling flow rate of the NanoScan SMPS was 0.75 L/min. Each measurement was 24 h long and performed in triplicate.

Statistical comparisons were performed using two-way analysis of variance (ANOVA) to evaluate the effects of electric motor model and power setting on total particle number concentration. Tukey–Kramer HSD was applied post hoc to identify statistically distinct groups while accounting for multiple comparisons. Although no formal tests for normality or homogeneity of variances were conducted, replicate measurements showed relatively consistent standard deviations across groups (see Table 1 and Table S2), and no clear violations of ANOVA assumptions were observed. Total particle number concentration was used as a univariate response variable to summarize emissions, which is appropriate for characterizing exposure potential.

## Results and discussion

In this study, we tested seven different electric motors at their maximum and minimum power operating settings and characterized the aerosol size distributions (Table 1).

**Table 1:** Descriptive statistics for the total number concentration and mode diameter of size distributions of aerosol particles emitted by the electric motors of household appliances, averaged across three measurements at the same power setting. The standard deviation is presented in parentheses. AP stands for “appliance” and symbolizes the electric motor from each household appliance

AP Model	Power Setting	Average Total Number Concentration (#/cm^3^) ^1^	Average Mode Diameter (nm) ^1^
AP A	Minimum Setting	38.5 (± 16.8)	49.6 (± 17.5)
AP A	Maximum Setting	809.6 (± 463.0)	43.7 (± 2.2)
AP B	Minimum Setting	1.3 (± 1.3)	26.2 (± 20.8)
AP B	Maximum Setting	6.6 (± 1.4)	75.6 (± 7.7)
AP C	Minimum Setting	2.0 (± 0.3)	55.9 (± 1.6)
AP C	Maximum Setting	268.9 (± 74.2)	53.0 (± 3.0)
AP D	Minimum Setting	14.5 (± 6.5)	55.2 (± 2.8)
AP D	Maximum Setting	179.3 (± 48.5)	51.7 (± 2.9)
AP E	Minimum Setting	3.7 (± 2.4)	42.8 (± 7.8)
AP E	Maximum Setting	652.0 (± 102.2)	41.7 (± 5.6)
AP F	Minimum Setting	30.5 (± 32.9)	48.3 (± 4.3)
AP F	Maximum Setting	228.6 (± 100.8)	42.8 (± 6.9)
AP G	Minimum Setting	1.0 (± 0.9)	43.6 (± 28.6)
AP G	Maximum Setting	1.7 (± 0.3)	57.6 (± 11.6)

1 Based on three measurements under the same conditions

**Fig. 2:**
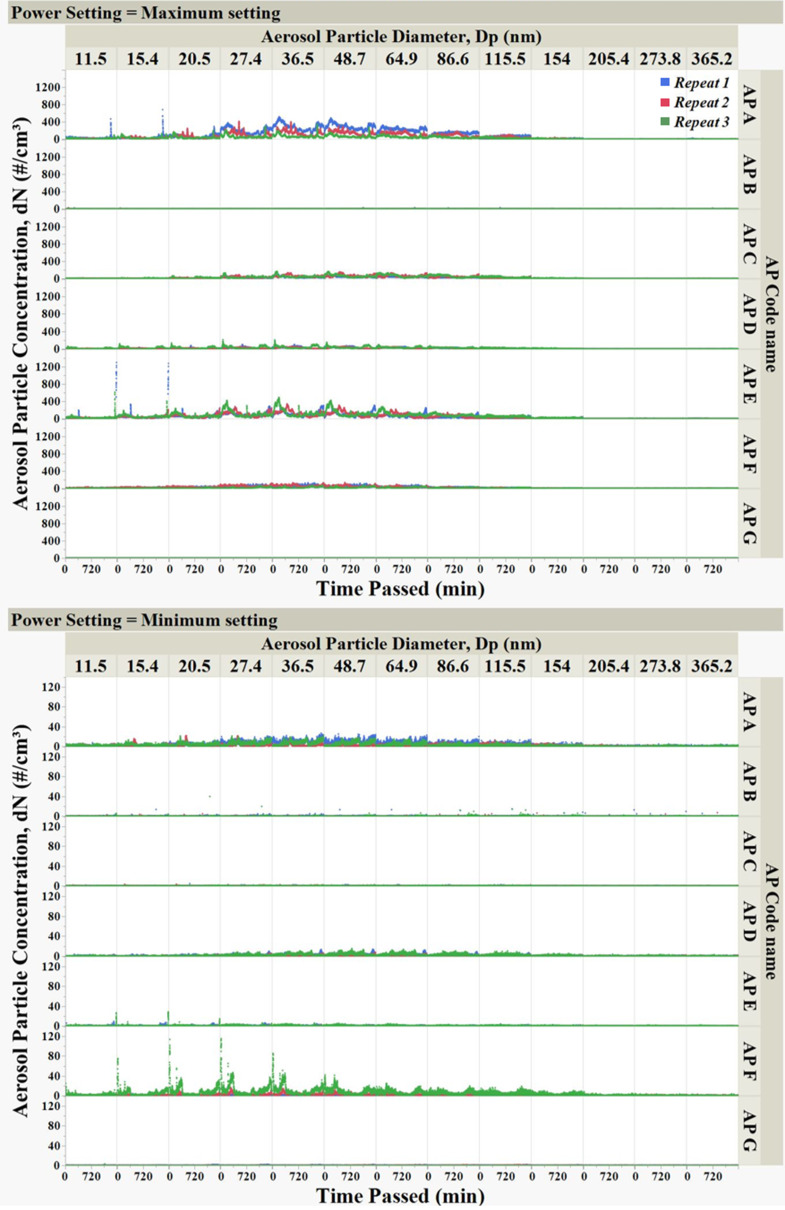
Emission of aerosol particles from electric motors of household appliances. Each measurement was 1440 min long, shown as the change in number concentration in each size channel, at the minimum and maximum-power settings for each electric motor. Data for three measurements plotted

The total number concentration never surpassed *4*000 cm^− 3^ at the maximum power and stayed below *5*00 cm^− 3^ at the minimum power settings (Fig. 2).

The range of nanoparticles emitted into particle-free clean air flowing past each motor at 40 SL/min in our experiments was similar to the particle number concentration in indoor areas during non-cooking hours reported in another study previously (Yu et al. 2015). Any emissions from electric motors operating indoors are added to the background nanoparticles. Our observations revealed higher nanoparticle concentrations at maximum power, while lower concentrations were observed at lower power, as depicted in Fig. 2, which was expected.

Total aerosol concentrations at the maximum power from electric motors AP A (809.6 ± 463.0 cm^− 3^) and AP E (652.0 ± 102.2 cm^− 3^) were observed to be the highest, while at the minimum setting electric motors AP A (38.5 ± 16.8 cm^− 3^) and AP F (30.5 ± 32.9 cm^− 3^) produced the highest emissions (Table 1).

**Fig. 3:**
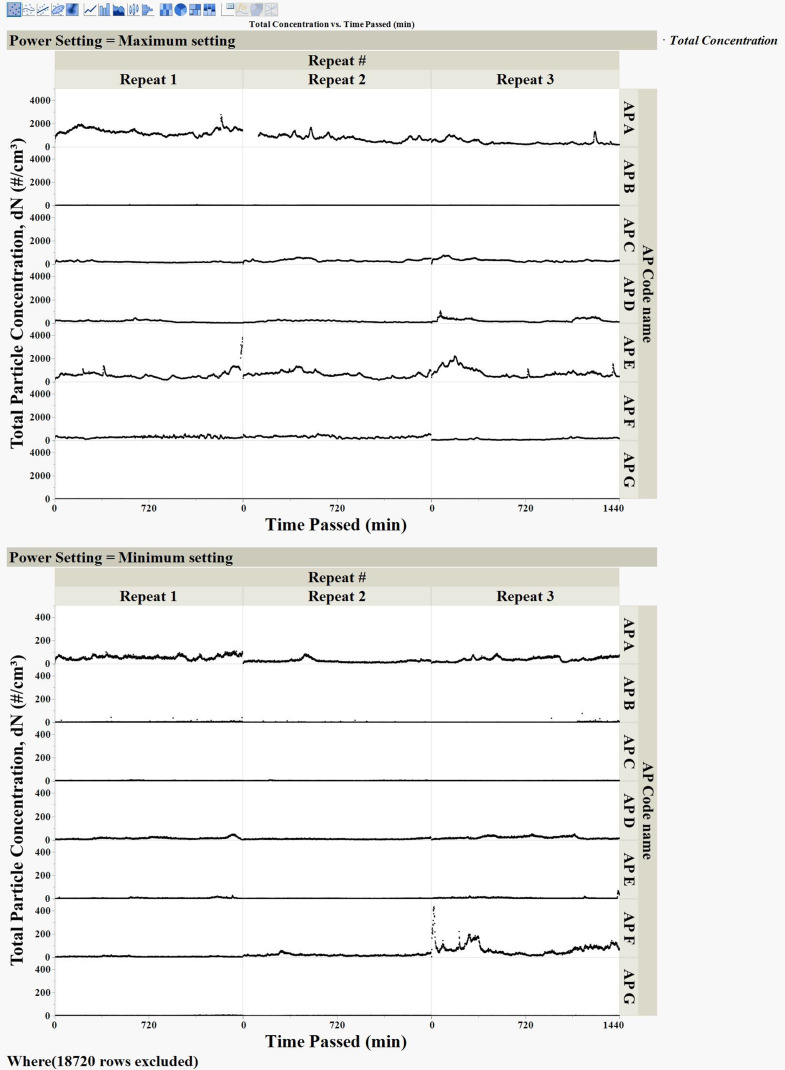
Emission of aerosol particles from electric motors of household appliances. Each measurement was 1440 min, shown as the total number of particles for all measured aerosol particle sizes, at the minimum and maximum-power settings for each electric motor. Data for three measurements plotted

At the maximum power setting, two of the motors (AP A and AP E) produced the most nanoparticles compared to all other motors, and nanoparticle emission started immediately upon starting the motors (Figs. 2 and 3). Their peak particle number concentrations observed—over 2700 particles cm⁻³—fall within or above the range reported for indoor environments with active sources. These values approach or surpass commonly cited background levels for occupied indoor spaces with increased inhalation exposure, which are typically in the range of 10³–10⁴ particles cm⁻³ (Wu et al. 2012); (Lowther et al. 2020). Electric motor AP E ranked second in total particle emission operating at maximum power– its total emissions were roughly 170 times higher compared to the minimum power setting. The total number concentration spiked to 3980 particles cm^− 3^ during the operation of electric motor AP E at maximum power, although the concentration decreased after several hours of operation (Fig. 3). Overall, despite the clear differences in emission levels of electric motors AP A and AP E at the two power settings, the average median and mode particle size were approximately 30–70 nm at both power settings (Table S2), indicating a similarity of nanoparticle size distributions.

A Two-Way ANOVA test was performed to determine the statistical differences between the levels of total aerosol particle emission from different motors at different power (Table S3). To investigate the effects of different parameter interactions, a post-hoc Tukey-Kramer Honestly Significant Difference (HSD) statistical analysis was performed. Table 2 shows the connecting letters report of Tukey-Kramer HSD, comparing the effect on the average total number concentrations of each of the 14 different combinations of 7 electric motors and 2 power settings.

**Table 2:** Connecting letters report of Tukey-Kramer HSD. Analysis was performed for combinations of electric motor model and power setting with average total number concentration. combinations that share no letters are different in a statistically significant manner

**Comparisons for all pairs using Tukey-Kramer HSD**									
**Confidence Quantile**									
q*	**Alpha**								
3.35377	0.05								
**Connecting Letters Report**									
**Level**									**Mean**
AP A Maximum setting	A								809.59547
AP E Maximum setting		B							651.93734
AP C Maximum setting			C						268.93076
AP F Maximum setting				D					228.63464
AP D Maximum setting					E				179.30254
AP A Minimum setting						F			38.55337
AP F Minimum setting						F			30.52308
AP D Minimum setting							G		14.50899
AP B Maximum setting							G	H	6.55781
AP E Minimum setting							G	H	3.75032
AP C Minimum setting								H	1.96770
AP G Maximum setting								H	1.68803
AP B Minimum setting								H	1.30953
AP G Minimum setting								H	1.02812

Levels not connected by same letter are significantly different

Through this analysis, average values which differ from one another in a statistically significant manner are revealed. Results are presented in the Connecting Letters Report and Ordered Differences Report. The Connecting Letters Report assigns a letter to all electric motor and power setting combinations. Combinations that share no letters in the connecting letters report have statistically significant different average values for total number concentration. The Ordered Differences Report compares all pairwise combinations of different motors and power settings.

Two-Way ANOVA analysis demonstrated that the model of electric motor, operating power setting, repeat/measurement number, and elapsed time all have significant effects on the total emission at *p* < 0.05, where the impact of each parameter on total emissions depends on the other parameters (Table S3). Post-hoc Tukey-Kramer HSD analysis confirmed the average total number concentration from APs A, C, D, E, and F at the maximum setting was significantly different from all other motor/power setting scenarios (Table 2). At the same time, the average total number concentrations emitted by APs A and F at the minimum setting were observed to be similar to each other, but statistically distinct from all other motor and power setting combinations. All motor/power setting scenarios when the average total number concentration was below 10 cm^− 3^ were statistically similar to each other (Table 2). The Ordered Differences Report, which provides a detailed comparison between all motor and power setting combinations, shows significant differences in emission between most of the combinations (Table 2). The two-way ANOVA results confirmed statistically significant effects of motor model, power setting, measurement repeat, and elapsed time on total particle emissions (*p* < 0.05), with interactions between parameters. Tukey–Kramer HSD analysis grouped conditions into statistically distinct emission levels, supporting the identification of consistently low- and high-emitting motors. Our results, with a novel focus on household appliance emissions and indoor air quality, support the conclusions in existing literature regarding the significance of electric motor model and operating power setting (Zarma et al. 2019; Goman et al. 2020).

**Fig. 4:**
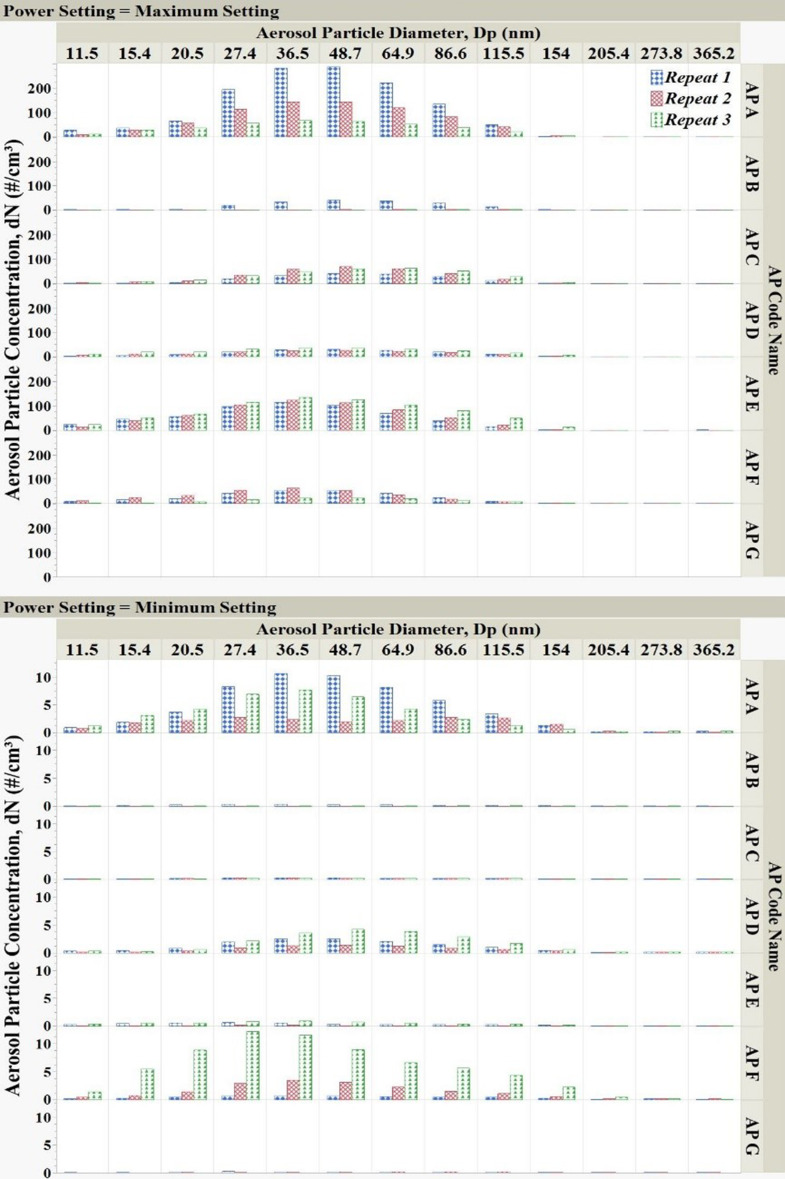
Emission of aerosol particles from electric motors of household appliances, shown as the overall average aerosol number concentration in each size channel at the minimum and maximum-power settings for each electric motor. Each measurement was 1440 min long. Data for three measurements plotted

The particle size distribution and concentration varied among different motors and also between different measurements with the same motor. The modes of the aerosol size distributions lie within the nanoparticle range (30–70 nm). For example, as shown in Table S2, the modes of size distributions during each measurement were observed to be significantly distinct, illustrating the variability of size distributions. Total aerosol emissions from the electric motors did not follow any consistent trend, as indicated by the high standard deviations in almost all measurements (Table S2). For all tested electric motors, except motor G—which emitted less than 2 aerosol particles per cm^3^ at both settings—the average total number concentration across three measurements varied significantly for each power setting (Table 1). Expectedly, most of the motors emitted higher total particle counts while operating at maximum power, and total emission was found to be, on average, 5–175 times greater than at minimum power operating settings. To illustrate these variations, data for each electric motor were plotted separately according to their maximum and minimum power settings (Figs. 2, 3 and 4).

Fewer fine particles (100–421.7 nm) were emitted by electric motors compared to the number of nanoparticles emitted (Fig. 4). Specifically, the average number of emitted particles across all measurements was 18.8 ± 43.6 cm^− 3^ in the 10–100 nm range (nanoparticles), compared to 2.0 ± 7.5 cm^− 3^ in the 100–421.7 nm range (fine particles). In this study, the majority (91%) of aerosol emissions were observed to fall in the nanoparticle size range, while only a minority (9%) of aerosol emissions were of particles larger than 100 nm. Despite high standard deviations and noticeable variability across measurements, the average mode of aerosol size distributions for all tested electric motors ranged from 20 nm to 80 nm in each measurement. This pattern shows a predominance of particles smaller than 100 nm in electric motor emissions, highlighting the need to focus attention on the potential health risks associated with nanoparticle exposure when dealing with the potential hazard of PM emissions from electric motors in household appliances. The observed variations in aerosol particle emissions imply that the mechanisms of particle generation by different motors are likely more complex than mechanical friction or heating. Direct mechanistic analysis was beyond the scope of this work, but future work should incorporate thermal imaging, wear analysis, and comparisons between brushed and brushless motor types to identify emission sources more precisely. The location of the electric motor in the design of each household appliance and the configuration of the cooling system may affect the emission of aerosol particles from a given electric motor. This high variability in aerosol particle emissions during the operation of most motors indicates the need for thorough testing and consideration during the design of new motors.

Recognizing the importance of transitioning away from combustion sources due to the adverse health effects linked to both indoor and outdoor air pollution exposure, it is important to address the contribution of non-combustion sources, particularly electric appliances, to indoor air pollution. Beyond household appliances, indoor electric vehicles are widespread– used in underground metro train networks, airport terminals, and beyond. The emission of airborne nanoparticles from motors in indoor electric vehicles and the corresponding emission mitigation strategies should be the subject of more extensive research. Considering that certain electric motors demonstrate minimal aerosol particle emissions, manufacturers have a choice of low-emission motors and are encouraged to adopt low-emission motor designs to mitigate particle emissions from their products. Alternatively, while not evaluated in this study, HEPA-grade filters placed downstream of the motor airflow path—similar to those used in vacuum cleaner exhaust systems—could serve as a practical mitigation strategy to reduce indoor nanoparticle emissions. On the advocacy and policy fronts, promoting awareness among industrial customers choosing electric motors for their appliances and the end consumers about the importance of choosing low-emission appliances can drive the market demand for cleaner electric motor technologies. This, coupled with more stringent regulations and incentives for manufacturers, can accelerate the adoption of safer electric motors, ultimately leading to healthier indoor environments.

## Conclusions

Many models of electric motors in household appliances are a significant source of nanoparticulate pollution indoors. All investigated electric motors emitted aerosol particles in the 10 nm to 421.7 nm size range, with peak concentrations emitted in the nanoparticulate size range (below 100 nm). Most of the motors emitted the highest aerosol particle numbers at maximum power operating settings, and significant differences in aerosol emission were observed with different electric motors. Our finding of the peak emission in the nanoparticulate size range is noteworthy because aerosol measurement instruments that are most commonly used to monitor aerosol particles in indoor air cannot detect nanoparticles, so the significance of the problem of particulate indoor air pollution generated by electric motors in household appliances had not been discovered and investigated to date. Nanoparticles have been linked to adverse health effects along with larger, fine and coarse aerosol particles, so many electric motors operated indoors present a potential health hazard that merits further investigation in terms of the effect on indoor air quality and the consequent adverse effects on wellbeing and health.

Designs of household appliances with electric motors that minimize aerosol particle emissions can reduce and even completely eliminate nanoparticle and fine particle emissions from electric motors in household appliances. The task can be accomplished by using electric motors that emit fewer or do not emit any aerosol particles, by sealing electric motors, or by installing HEPA filters downstream of electric motors in appliances, particularly into the cooling air outlet. The latter approach, although speculative, is consistent with filtration strategies used in devices such as vacuum cleaners and HVAC systems and could serve as a practical mitigation option to reduce indoor nanoparticle emissions. We expect that our findings will trigger more research into the discovered problem of nanoparticle and fine aerosol particle indoor air pollution from electric motors in household appliances. While it was impossible to investigate all types of electric motors at all power settings in the vast number of household appliances on the global market today, this study demonstrated clearly that some electric motors emit a very significant number of aerosol particles while others hardly emit any. The finding of high emissions from some motors identified the problem, while the finding of negligible emissions from other motors shows that it is possible to solve it. It is now the job of electric motor and appliance designers and manufacturers to consider monitoring ultrafine and fine aerosol particle emissions from their devices and implement engineering solutions to mitigate the problem.

This study focuses on characterizing emissions from a manageable selection of electric motors in order to discover the phenomenon as such. Thousands of electric motor types are found in household appliances, and a representative comprehensive study of all of them, as well as mechanistic, chemical composition, and design-specific investigations, are left to future research.

In addition to electric motors in household appliances, due to the high power, electric motors in elevators and indoor electric vehicles, such as those used in underground metro train networks and airport terminals, must be investigated more thoroughly as a source of nanoparticulate indoor air pollution. Concurrently with these observational studies, mitigation strategies to reduce nanoparticulate electric motor emissions from indoor electric vehicles should be developed and studied.

## Electronic supplementary material

Below is the link to the electronic supplementary material.

Supplementary Material 1

## Data Availability

The datasets generated during and/or analyzed during the current study are not publicly available because the researchers are still conducting follow-up analyses and wish to control when and how data are released, but they are available from the corresponding author upon reasonable request.
