# Supplementary material for: Emission of airborne nanoparticles from electric motors of household appliances
Source: Air Qual Atmos Health. 2025 May 13;18(7):1945–55. doi: 10.1007/s11869-025-01744-1 (PMC12343712; doi:10.1007/s11869-025-01744-1)
Supplement: Supplementary file 1 — Supplementary Material 1 [file 11869_2025_1744_MOESM1_ESM.pdf]

Supplementary Information

Emission of Airborne Nanoparticles from Electric Motors of Household Appliances

Yevgen Nazarenko[*,1], Elliot Zolfaghar[2], Devendra Pal[3], Léa Quellard[3], and Parisa A. Ariya[*,2,3]

[1] Department of Environmental and Public Health Sciences, University of Cincinnati, 160 Panzeca Way, Cincinnati, OH 45267, USA.

[2] Department of Chemistry, McGill University, 801 Sherbrooke Street West, Montréal, QC H3A 2K6, Canada.

[3] Department of Atmospheric and Oceanic Sciences, McGill University, 805 Sherbrooke Street West, Montreal, QC H3A 0B9, Canada.

*Corresponding authors:

1. Parisa A. Ariya, Phone: (514) 398-6931 & (514) 398-3615, Fax: (514) 398-3797, Email:parisa.ariya@mcgill.ca

2. Yevgen Nazarenko, Phone: (514) 431-4115 & (513) 558-2883, Email:nazareyn@ucmail.uc.edu

Table of Contents

Supplementary Information 1
Table of Contents 2
Supplemental Figures and Tables 3

Supplemental Figures and Tables

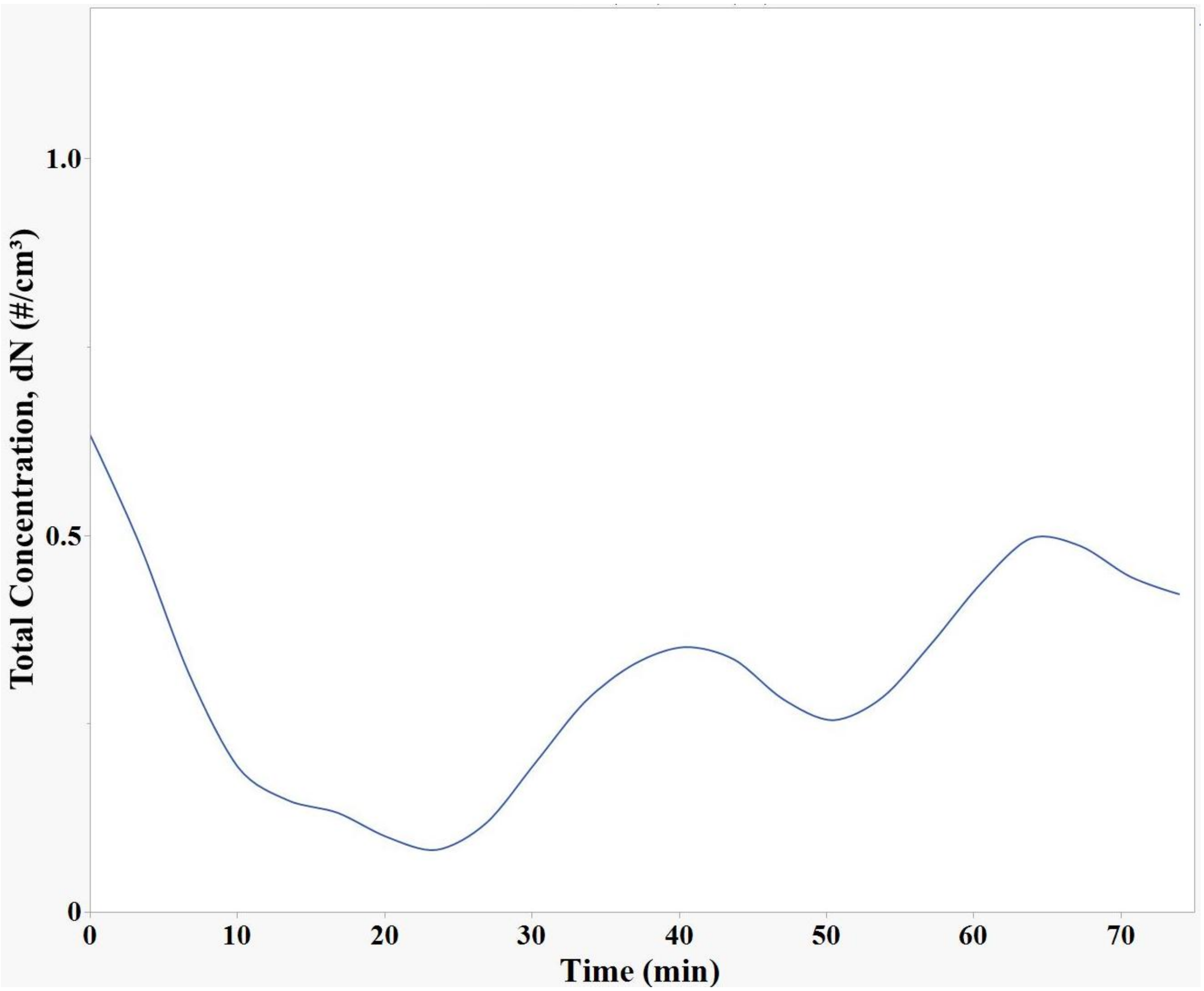

Figure S1. Background clean air aerosol concentration in the chamber measured using the NanoScan SMPS instrument.

Table S1. Particle size measurement channels of the NanoScan SMPS instrument.

Geometric Midpoint of the Particle Size Channel, Dp	Particle Size Range
11.5 nm	10.0 nm - 13.3 nm
15.4 nm	13.3 nm - 17.8 nm
20.5 nm	17.8 nm - 23.7 nm
27.4 nm	23.7 nm - 31.6 nm
36.5 nm	31.6 nm - 42.2 nm
48.7 nm	42.2 nm - 56.2 nm

64.9 nm	56.2 nm - 75.0 nm
86.6 nm	75.0 nm - 100.0 nm
115.5 nm	100.0 nm - 133.4 nm
154 nm	133.4 nm - 177.8 nm
205.4 nm	177.8 nm - 237.1 nm
273.8 nm	237. nm 1 - 316.2 nm
365.2 nm	316.2 nm - 421.7 nm

Table S2. Descriptive statistics for the size distributions of aerosol particles emitted by the electric motors of household appliances. GSD stands for "geometric standard deviation". The standard deviation is presented in parentheses. AP stands for "appliance" and symbolizes the electric motor from each household appliance.

AP Code	Repeat/ Measurement #	Power Setting	Average Total Number concentration (#/cm^3)	Average Median (nm)	Average Mean (nm)	Average Mode (nm)	Average GSD
AP A	Measurement 1	Minimum Setting	55.1 (±15.7)	46.3 (±5.9)	55.9 (±7.0)	44.9 (±15.6)	1.8 (±0.1)
AP A	Measurement 2	Minimum Setting	21.4 (±12.5)	60.4 (±22.3)	69.0 (±16.6)	68.9 (±37.4)	2.1 (±0.2)
AP A	Measurement 3	Minimum Setting	39.1 (±16.5)	36.9 (±6.8)	48.5 (±8.3)	34.9 (±14.6)	1.9 (±0.2)
AP A	Measurement 1	Maximum setting	1302.5 (±278.4)	45.7 (±4.6)	50.0 (±4.6)	43.9 (±7.2)	1.6 (±0.1)
AP A	Measurement 2	Maximum setting	742.6 (±241.6)	46.0 (±7.1)	51.0 (±6.5)	45.9 (±11.5)	1.7 (±0.1)
AP A	Measurement 3	Maximum setting	383.7 (±201.1)	42.3 (±5.6)	49.0 (±5.0)	41.5 (±10.9)	1.8 (±0.1)
AP B	Measurement 1	Minimum Setting	2.7 (±2.6)	49.3 (±31.6)	71.3 (±32.7)	49.3 (±47.1)	2.1 (±0.6)
AP B	Measurement 2	Minimum Setting	0.1 (±0.8)	8.7 (±36.4)	10.3 (±36.2)	8.9 (±40.4)	0.2 (±0.6)
AP B	Measurement 3	Minimum Setting	1.0 (±3.1)	18.8 (±36.7)	22.6 (±41.7)	20.3 (±43.6)	0.5 (±0.9)
AP B	Measurement 1	Maximum setting	8.1 (±4.3)	66.2 (±18.7)	81.5 (±18.6)	68.9 (±31.2)	1.9 (±0.3)

AP B	Measurement 2	Maximum setting	6.4 (±3.3)	68.3 (±24.8)	83.6 (±22.3)	73.8 (±39.9)	2.0 (±0.3)
AP B	Measurement 3	Maximum setting	5.2 (±2.0)	78.0 (±26.5)	92.2 (±22.5)	84.0 (±42.8)	2.0 (±0.4)
AP C	Measurement 1	Minimum Setting	2.2 (±1.3)	55.9 (±29.6)	78.0 (±28.4)	56.5 (±46.1)	2.2 (±0.5)
AP C	Measurement 2	Minimum Setting	2.0 (±1.3)	54.9 (±33.6)	75.8 (±33.1)	54.2 (±47.3)	2.1 (±0.6)
AP C	Measurement 3	Minimum Setting	1.7 (±1.0)	58.4 (±35.5)	80.5 (±34.5)	57.2 (±48.8)	2.1 (±0.6)
AP C	Measurement 1	Maximum setting	183.6 (±52.2)	54.5 (±5.6)	58.7 (±5.2)	54.9 (±11.4)	1.6 (±0.1)
AP C	Measurement 2	Maximum setting	304.9 (±106.3)	50.3 (±4.8)	54.4 (±4.6)	49.5 (±8.1)	1.6 (±0.1)
AP C	Measurement 3	Maximum setting	318.4 (±121.3)	53.6 (±10.6)	58.7 (±8.6)	54.5 (±16.5)	1.7 (±0.1)
AP D	Measurement 1	Minimum Setting	14.4 (±7.8)	50.1 (±13.9)	63.6 (±14.4)	52.0 (±24.8)	1.9 (±0.2)
AP D	Measurement 2	Minimum Setting	8.0 (±3.2)	55.4 (±15.1)	72.4 (±17.2)	56.7 (±28.2)	2.0 (±0.3)
AP D	Measurement 3	Minimum Setting	21.1 (±9.6)	55.8 (±11.3)	66.9 (±11.3)	57.0 (±22.4)	1.8 (±0.2)
AP D	Measurement 1	Maximum setting	152.4 (±86.9)	51.0 (±8.3)	56.5 (±7.6)	54.4 (±17.8)	1.8 (±0.1)
AP D	Measurement 2	Maximum setting	150.2 (±70.3)	44.6 (±5.6)	51.3 (±5.3)	48.5 (±17.6)	1.9 (±0.1)
AP D	Measurement 3	Maximum setting	235.4 (±141.7)	49.0 (±15.3)	55.3 (±13.8)	52.1 (±21.1)	1.8 (±0.1)
AP E	Measurement 1	Minimum Setting	4.3 (±3.4)	43.0 (±23.9)	64.3 (±25.3)	41.3 (±36.8)	2.2 (±0.5)
AP E	Measurement 2	Minimum Setting	1.1 (±0.9)	38.9 (±35.8)	59.8 (±41.6)	35.8 (±40.8)	1.8 (±0.9)
AP E	Measurement 3	Minimum Setting	5.8 (±5.8)	51.2 (±25.2)	69.5 (±24.4)	51.2 (±37.5)	2.2 (±0.3)
AP E	Measurement 1	Maximum setting	570.2 (±361.5)	38.9 (±4.9)	44.2 (±5.0)	37.9 (±7.1)	1.7 (±0.1)
AP E	Measurement 2	Maximum setting	619.2 (±245.7)	40.1 (±8.4)	45.5 (±7.3)	39.2 (±11.7)	1.7 (±0.1)

AP E	Measurement 3	Maximum setting	766.5 (±396.0)	44.8 (±6.2)	52.0 (±6.2)	48.1 (±17.8)	1.8 (±0.1)
AP F	Measurement 1	Minimum Setting	5.5 (±2.6)	52.4 (±21.0)	71.5 (±20.7)	53.3 (±34.2)	2.2 (±0.3)
AP F	Measurement 2	Minimum Setting	18.3 (±7.7)	45.9 (±10.0)	59.4 (±12.0)	46.0 (±21.2)	1.9 (±0.2)
AP F	Measurement 3	Minimum Setting	67.8 (±51.0)	44.0 (±12.1)	55.4 (±11.0)	45.5 (±25.2)	1.9 (±0.2)
AP F	Measurement 1	Maximum setting	272.3 (±74.4)	41.6 (±2.6)	46.7 (±3.1)	41.9 (±6.3)	1.7 (±0.1)
AP F	Measurement 2	Maximum setting	300.2 (±92.2)	36.5 (±2.1)	41.8 (±2.8)	36.3 (±3.9)	1.7 (±0.1)
AP F	Measurement 3	Maximum setting	113.3 (±67.5)	49.7 (±7.1)	56.3 (±6.8)	50.1 (±16.3)	1.7 (±0.1)
AP G	Measurement 1	Minimum Setting	1.9 (±1.3)	61.4 (±41.3)	80.4 (±38.6)	60.5 (±54.1)	2.1 (±0.7)
AP G	Measurement 2	Minimum Setting	1.1 (±0.9)	59.7 (±52.5)	74.1 (±54.6)	59.8 (±61.2)	1.5 (±1.0)
AP G	Measurement 3	Minimum Setting	0.1 (±0.3)	10.8 (±37.5)	13.5 (±40.0)	10.6 (±40.7)	0.3 (±0.7)
AP G	Measurement 1	Maximum setting	1.9 (±1.0)	60.6 (±33.8)	83.0 (±33.5)	61.2 (±50.6)	2.1 (±0.6)
AP G	Measurement 2	Maximum setting	1.9 (±1.0)	65.9 (±38.5)	85.9 (±33.7)	67.0 (±56.2)	2.2 (±0.5)
AP G	Measurement 3	Maximum setting	1.3 (±1.0)	47.1 (±38.4)	68.7 (±41.5)	44.6 (±47.3)	1.9 (±0.8)

Table S3. Results of the 2-Way ANOVA test. "N_{parm}" stands for "number of parameters. "DF" stands for "degrees of freedom".

Parameter(s)	Nparm	DF	Sum of Squares	F Ratio	Prob > F
**Model of Motor**	6	6	1322249569	11813.54	<.0001*
**Power Setting**	1	1	11303358573	69868.57	<.0001*
**Repeat #**	2	2	20140482	539.8310	<.0001*

**Elapsed Time (min)**	1	1	18170353.5	974.0501	<.0001*
**Model of Motor*Power Setting**	6	6	1186587079	10601.48	<.0001*
**Model of Motor*Repeat #**	12	12	337038311	1505.622	<.0001*
**Model of Motor*Elapsed Time (min)**	6	6	23215651	207.4185	<.0001*
**Power Setting*Repeat #**	2	2	26573665.1	712.2614	<.0001*
**Power Setting*Elapsed Time (min)**	1	1	19047201.7	1021.055	<.0001*
**Repeat #*Elapsed Time (min)**	2	2	6828410.25	183.0238	<.0001*

Table S4. Results of the Ordered Differences Report performed for comparison of pairs of electric motors and power settings with the average total number concentration. The values in the "Difference" column is the difference between the average total number concentration of the two pairs being compared. Standard Error of the Difference is the error associated with the difference between the two average total number concentrations. Lower and Upper CL are the lower and upper limits of the confidence interval for the difference between the two average total number concentrations. The p-value is a measure of the significance of the difference between the average total number concentrations for the pair being compared.

Ordered Differences Report

Pairwise Comparison Model of Motor*Power Setting	Pairwise Comparison Model of Motor*Power Setting	Difference	Lower CL	Upper CL	p-Value
AP A Maximum setting	AP G Minimum setting	808.5673	796.912	820.2231	<.0001*
AP A Maximum setting	AP B Minimum setting	808.2859	796.629	819.9430	<.0001*
AP A Maximum setting	AP G Maximum setting	807.9074	796.252	819.5625	<.0001*
AP A Maximum setting	AP C Minimum setting	807.6278	795.971	819.2848	<.0001*
AP A Maximum setting	AP E Minimum setting	805.8452	794.190	817.5002	<.0001*
AP A Maximum setting	AP B Maximum setting	803.0377	791.381	814.6947	<.0001*
AP A Maximum setting	AP D Minimum setting	795.0865	783.430	806.7429	<.0001*
AP A Maximum setting	AP F Minimum setting	779.0724	767.415	790.7294	<.0001*

Pairwise Comparison Model of Motor*Power Setting	Pairwise Comparison Model of Motor*Power Setting	Difference	Lower CL	Upper CL	p-Value
AP A Maximum setting	AP A Minimum setting	771.0421	759.387	782.6971	<.0001*
AP E Maximum setting	AP G Minimum setting	650.9092	639.252	662.5663	<.0001*
AP E Maximum setting	AP B Minimum setting	650.6278	638.969	662.2862	<.0001*
AP E Maximum setting	AP G Maximum setting	650.2493	638.593	661.9057	<.0001*
AP E Maximum setting	AP C Minimum setting	649.9696	638.311	661.6280	<.0001*
AP E Maximum setting	AP E Minimum setting	648.1870	636.531	659.8434	<.0001*
AP E Maximum setting	AP B Maximum setting	645.3795	633.721	657.0379	<.0001*
AP E Maximum setting	AP D Minimum setting	637.4283	625.771	649.0861	<.0001*
AP A Maximum setting	AP D Maximum setting	630.2929	618.635	641.9507	<.0001*
AP E Maximum setting	AP F Minimum setting	621.4143	609.756	633.0727	<.0001*
AP E Maximum setting	AP A Minimum setting	613.3840	601.728	625.0404	<.0001*
AP A Maximum setting	AP F Maximum setting	580.9608	569.304	592.6179	<.0001*
AP A Maximum setting	AP C Maximum setting	540.6647	529.008	552.3218	<.0001*
AP E Maximum setting	AP D Maximum setting	472.6348	460.976	484.2939	<.0001*
AP E Maximum setting	AP F Maximum setting	423.3027	411.644	434.9611	<.0001*
AP E Maximum setting	AP C Maximum setting	383.0066	371.348	394.6650	<.0001*
AP C Maximum setting	AP G Minimum setting	267.9026	256.245	279.5604	<.0001*
AP C Maximum setting	AP B Minimum setting	267.6212	255.962	279.2803	<.0001*
AP C Maximum setting	AP G Maximum setting	267.2427	255.586	278.8998	<.0001*
AP C Maximum setting	AP C Minimum setting	266.9631	255.304	278.6221	<.0001*
AP C Maximum setting	AP E Minimum setting	265.1804	253.523	276.8375	<.0001*
AP C Maximum setting	AP B Maximum setting	262.3729	250.714	274.0320	<.0001*
AP C Maximum setting	AP D Minimum setting	254.4218	242.763	266.0802	<.0001*
AP C Maximum setting	AP F Minimum setting	238.4077	226.749	250.0668	<.0001*
AP C Maximum setting	AP A Minimum setting	230.3774	218.720	242.0344	<.0001*
AP F Maximum setting	AP G Minimum setting	227.6065	215.949	239.2642	<.0001*
AP F Maximum setting	AP B Minimum setting	227.3251	215.666	238.9842	<.0001*
AP F Maximum setting	AP G Maximum setting	226.9466	215.290	238.6037	<.0001*
AP F Maximum setting	AP C Minimum setting	226.6669	215.008	238.3260	<.0001*
AP F Maximum setting	AP E Minimum setting	224.8843	213.227	236.5414	<.0001*
AP F Maximum setting	AP B Maximum setting	222.0768	210.418	233.7359	<.0001*
AP F Maximum setting	AP D Minimum setting	214.1256	202.467	225.7840	<.0001*
AP F Maximum setting	AP F Minimum setting	198.1116	186.452	209.7706	<.0001*
AP F Maximum setting	AP A Minimum setting	190.0813	178.424	201.7383	<.0001*
AP D Maximum setting	AP G Minimum setting	178.2744	166.616	189.9328	<.0001*
AP D Maximum setting	AP B Minimum setting	177.9930	166.333	189.6528	<.0001*
AP D Maximum setting	AP G Maximum setting	177.6145	165.957	189.2722	<.0001*
AP D Maximum setting	AP C Minimum setting	177.3348	165.675	188.9946	<.0001*
AP D Maximum setting	AP E Minimum setting	175.5522	163.894	187.2099	<.0001*
AP D Maximum setting	AP B Maximum setting	172.7447	161.085	184.4045	<.0001*
AP D Maximum setting	AP D Minimum setting	164.7935	153.134	176.4526	<.0001*
AP A Maximum setting	AP E Maximum setting	157.6581	146.002	169.3145	<.0001*
AP D Maximum setting	AP F Minimum setting	148.7795	137.120	160.4392	<.0001*
AP D Maximum setting	AP A Minimum setting	140.7492	129.091	152.4069	<.0001*
AP C Maximum setting	AP D Maximum setting	89.6282	77.968	101.2880	<.0001*
AP F Maximum setting	AP D Maximum setting	49.3321	37.672	60.9918	<.0001*
AP C Maximum setting	AP F Maximum setting	40.2961	28.637	51.9552	<.0001*
AP A Minimum setting	AP G Minimum setting	37.5252	25.870	49.1810	<.0001*
AP A Minimum setting	AP B Minimum setting	37.2438	25.587	48.9009	<.0001*
AP A Minimum setting	AP G Maximum setting	36.8653	25.210	48.5204	<.0001*
AP A Minimum setting	AP C Minimum setting	36.5857	24.929	48.2427	<.0001*
AP A Minimum setting	AP E Minimum setting	34.8031	23.148	46.4581	<.0001*

Pairwise Comparison Model of Motor*Power Setting	Pairwise Comparison Model of Motor*Power Setting	Difference	Lower CL	Upper CL	p-Value
AP A Minimum setting	AP B Maximum setting	31.9956	20.339	43.6526	<.0001*
AP F Minimum setting	AP G Minimum setting	29.4950	17.837	41.1527	<.0001*
AP F Minimum setting	AP B Minimum setting	29.2135	17.554	40.8726	<.0001*
AP F Minimum setting	AP G Maximum setting	28.8350	17.178	40.4921	<.0001*
AP F Minimum setting	AP C Minimum setting	28.5554	16.896	40.2145	<.0001*
AP F Minimum setting	AP E Minimum setting	26.7728	15.116	38.4298	<.0001*
AP A Minimum setting	AP D Minimum setting	24.0444	12.388	35.7008	<.0001*
AP F Minimum setting	AP B Maximum setting	23.9653	12.306	35.6243	<.0001*
AP F Minimum setting	AP D Minimum setting	16.0141	4.356	27.6725	0.0004*
AP D Minimum setting	AP G Minimum setting	13.4809	1.824	25.1379	0.0080*
AP D Minimum setting	AP B Minimum setting	13.1995	1.541	24.8579	0.0108*
AP D Minimum setting	AP G Maximum setting	12.8210	1.165	24.4773	0.0161*
AP D Minimum setting	AP C Minimum setting	12.5413	0.883	24.1997	0.0215*
AP D Minimum setting	AP E Minimum setting	10.7587	-0.898	22.4151	0.1070
AP A Minimum setting	AP F Minimum setting	8.0303	-3.627	19.6873	0.5471
AP D Minimum setting	AP B Maximum setting	7.9512	-3.707	19.6096	0.5644
AP B Maximum setting	AP G Minimum setting	5.5297	-6.128	17.1874	0.9489
AP B Maximum setting	AP B Minimum setting	5.2483	-6.411	16.9074	0.9662
AP B Maximum setting	AP G Maximum setting	4.8698	-6.787	16.5268	0.9820
AP B Maximum setting	AP C Minimum setting	4.5901	-7.069	16.2492	0.9894
AP B Maximum setting	AP E Minimum setting	2.8075	-8.850	14.4645	0.9999
AP E Minimum setting	AP G Minimum setting	2.7222	-8.934	14.3779	1.0000
AP E Minimum setting	AP B Minimum setting	2.4408	-9.216	14.0978	1.0000
AP E Minimum setting	AP G Maximum setting	2.0623	-9.593	13.7173	1.0000
AP E Minimum setting	AP C Minimum setting	1.7826	-9.874	13.4397	1.0000
AP C Minimum setting	AP G Minimum setting	0.9396	-10.718	12.5973	1.0000
AP G Maximum setting	AP G Minimum setting	0.6599	-10.996	12.3156	1.0000
AP C Minimum setting	AP B Minimum setting	0.6582	-11.001	12.3172	1.0000
AP G Maximum setting	AP B Minimum setting	0.3785	-11.279	12.0356	1.0000
AP B Minimum setting	AP G Minimum setting	0.2814	-11.376	11.9391	1.0000
AP C Minimum setting	AP G Maximum setting	0.2797	-11.377	11.9367	1.0000
